# Prognostic value of early, conventional proton magnetic resonance spectroscopy in cooled asphyxiated infants

**DOI:** 10.1186/s12887-018-1269-6

**Published:** 2018-09-15

**Authors:** Hajnalka Barta, Agnes Jermendy, Marton Kolossvary, Lajos R. Kozak, Andrea Lakatos, Unoke Meder, Miklos Szabo, Gabor Rudas

**Affiliations:** 1 1st Department of Paediatrics, Semmelweis University, Budapest, Hungary; 2 MTA-SE Cardiovascular Imaging Research Group, Heart and Vascular Center, Semmelweis University, Budapest, Hungary; 3 MR Research Center, Semmelweis University, Budapest, Hungary

**Keywords:** Perinatal asphyxia, Hypoxic-ischemic encephalopathy, Proton magnetic resonance spectroscopy, Conventional sequence, Neurodevelopmental outcome

## Abstract

**Background:**

Neonatal hypoxic-ischemic encephalopathy (HIE) commonly leads to neurodevelopmental impairment, raising the need for prognostic tools which may guide future therapies in time. Prognostic value of proton MR spectroscopy (H-MRS) between 1 and 46 days of age has been extensively studied; however, the reproducibility and generalizability of these methods are controversial in a general clinical setting. Therefore, we investigated the prognostic performance of conventional H-MRS during first 96 postnatal hours in hypothermia-treated asphyxiated neonates.

**Methods:**

Fifty-one consecutive hypothermia-treated HIE neonates were examined by H-MRS at three echo-times (TE = 35, 144, 288 ms) between 6 and 96 h of age, depending on clinical stability. Patients were divided into favorable (*n* = 35) and unfavorable (*n* = 16) outcome groups based on psychomotor and mental developmental index (PDI and MDI, Bayley Scales of Infant Development II) scores (≥ 70 versus < 70 or death, respectively), assessed at 18–26 months of age. Associations between 36 routinely measured metabolite ratios and outcome were studied. Age-dependency of metabolite ratios in whole patient population was assessed. Prognostic performance of metabolite ratios was evaluated by Receiver Operating Characteristics (ROC) analysis.

**Results:**

Three metabolite ratios showed significant difference between outcome groups after correction for multiple testing (*p* < 0.0014): myo-inositol (mIns)/N-acetyl-aspartate (NAA) height, mIns/creatine (Cr) height, both at TE = 35 ms, and NAA/Cr height at TE = 144 ms. Assessment of age-dependency showed that all 3 metabolite ratios (mIns/NAA, NAA/Cr and mIns/Cr) stayed constant during first 96 postnatal hours, rendering them optimal for prediction. ROC analysis revealed that mIns/NAA gives better prediction for outcome than NAA/Cr and mIns/Cr with cut-off values 0.6798 0.6274 and 0.7798, respectively, (AUC 0.9084, 0.8396 and 0.8462, respectively, *p* < 0.00001); mIns/NAA had the highest specificity (95.24%) and sensitivity (84.62%) for predicting outcome of neonates with HIE any time during the first 96 postnatal hours.

**Conclusions:**

Our findings suggest that during first 96 h of age even conventional H-MRS could be a useful prognostic tool in predicting the outcome of asphyxiated neonates; mIns/NAA was found to be the best and age-independent predictor.

**Electronic supplementary material:**

The online version of this article (10.1186/s12887-018-1269-6) contains supplementary material, which is available to authorized users.

## Background

Perinatal asphyxia and consequential hypoxic-ischemic encephalopathy (HIE) remains one of the leading causes of perinatal brain injury, affecting more than two million neonates yearly worldwide [1]. Although full recovery is possible, HIE can also lead to permanent mental or psychomotor disability [2].

Currently, therapeutic hypothermia is the one and only neuroprotective method proven effective to reduce mortality and long-term morbidity in HIE [3]. However, mortality or moderate to severe developmental delay still affects over 40% of cooled infants, demanding future therapeutic approaches additional to hypothermia [4, 5]. In theory, the key to successful neuroprotection is the earliest possible initiation regardless of the therapy chosen [6]. This in turn requires proper and timely diagnosis and early establishment of prognosis [7].

This underscores the need for an appropriate and as-early-as-possible prognostic tool for the selection of infants who are most likely to suffer moderate to severe disability and would thus benefit from future personalized neuroprotective protocols.

Proton magnetic resonance spectroscopy (H-MRS) is one of the tools proposed for such a biomarker [8]. This examination is becoming increasingly widespread in various medical fields, i.e. tumor diagnosis or neurodegenerative diseases. H-MRS usually accompanies brain magnetic resonance imaging (MRI) scans, and is capable of registering the spectra of various metabolites present in the examined volume of interest (VOI). Since water is the molecule most abundantly present in brain tissue, its acquired spectrum would be several orders of magnitude higher than those of other metabolites; consequently, acquisition of H-MRS requires suppression of the water signal. This can be achieved by several acquisition protocols [9]. The analysis of the acquired spectrum informs the clinician of the metabolic state of the examined tissue, providing valuable functional information in a non-invasive way. To acquire motionless images during brain MR scans, most infants require sufficient sedation and intravenous access, not all; however, no administration of contrast material is necessary.

Several studies investigated the prognostic power of H-MRS in neonatal asphyxia, between 4 h and 46 days of age [10–19], often covering a wide age range, given the need for earliest possible prognosis.

Establishing the reproducibility of H-MRS as a prognostic biomarker also poses a problem [20], as previous studies used a wide range of data-optimizing equipment, software, or absolute quantification approaches to improve data quality. Taken together, there is no universal agreement regarding how H-MRS should be applied in the daily clinical practice.

### Aim

The purpose of our study was to determine the prognostic value of a completely conventional H-MRS sequence (i.e. without special equipment and post-processing techniques other than basic vendor-provided analysis), performed before the 96th hour of life in infants with HIE, analyzing various metabolite ratios, their age-dependence and association with long-term neurodevelopmental outcome.

## Methods

### Patient selection

In our retrospective descriptive analysis, we reviewed all 283 patients with suspected HIE born between January 2006 and December 2010 and admitted to the regional cooling center, the Neonatal Intensive Care Unit (NICU) of the 1st Department of Paediatrics, Semmelweis University, Budapest, Hungary.

From this patient pool, we only included patients who (A) fulfilled the diagnostic criteria for moderate to severe HIE according to the international TOBY trial [21], being as follows: (i) at least one of the following: continued need for resuscitation/ventilation at 10 min after birth, OR Apgar score ≤ 5 at 5 min after birth OR pH < 7.0 or BE ≤ − 16 mmol/L within 60 min after birth AND (ii) altered level of consciousness (lethargy, stupor or coma) AND hypotonia or abnormal reflexes or seizures AND (iii) abnormal brain background activity registered on amplitude-integrated electroencephalography (aEEG). Additional inclusion criteria were (B) brain H-MRS scan performed before the 96th postnatal hour AND (C) having a neurodevelopmental follow-up examination using the Bayley Scales of Infant Development II between 18 and 26 months of age, as detailed below OR death (< 28 days of age OR > 28 days associated with HIE).

We excluded all patients who (a) had other underlying conditions, which could be responsible for encephalopathy besides asphyxia (i.e. stroke, intracranial hemorrhage, congenital malformation or metabolic disease). As only early onset (< 6 postnatal hours) hypothermia treatment was thought to be neuroprotective at the time of the study, we excluded patients who (b) did not receive hypothermia treatment due to delayed admission. Further exclusion criteria were: (c) gestational age < 36 weeks and (d) low quality brain H-MRS.

**Fig. 1:**
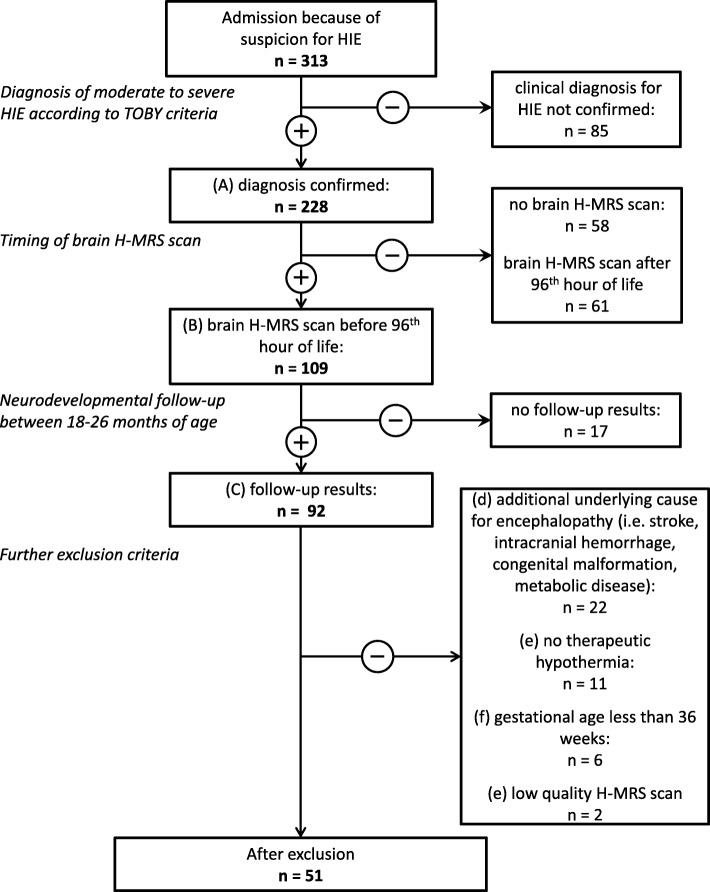
Inclusion and exclusion criteria

Altogether, 51 patients met inclusion criteria and were included in the analysis (Fig. 1).

### Clinical care

Whole-body hypothermia treatment was initiated as soon as possible but within 6 h after delivery, using a water-filled mattress (Tecotherm©; TecCom, Halle, Germany). The target rectal temperature was between 33 and 34 °C, maintained for 72 h. In the rewarming phase, temperature increase velocity was 0.5 °C/h. All infants were ventilated throughout the cooling and rewarming phase.

Continuous morphine (Morph. hydrochlor. 10 mg/mL; TEVA Magyarország Zrt., Gödöllő, Hungary) sedation (10 μg/kg BW/h) was started following the loading dose (0.1 mg/kg BW) administered when the cooling was initiated. Phenobarbitone (Gardenal 40 mg; Aventis, Maisons-Alfort, France, 20 mg/kg BW) was given as the first line of anticonvulsant therapy if clinical or electrophysiological seizures were detected. In case of noncontrolled seizures, the phenobarbitone loading dose was repeated, or midazolam (Midazolam Torrex 5 mg/ml; Chiesi Pharmaceuticals GmbH, Vienna, Austria) was given in single or repeated doses (0.1 mg/kg BW) or in continuous infusion (0.1 mg/kg BW/h). In some cases, newborns received lidocain, phenytoin, diazepam or chloral hydrate alternatively, according to the attending clinician’s decision.

The severity of encephalopathy was determined based on a combination of aEEG background activity at 6 h of age and Sarnat staging at admission [22]. Infants with abnormal aEEG pattern by 6 h of age (burst suppression (BS), low voltage (LV) or flat trace (FT)) OR meeting Sarnat stage 3 criteria were considered having severe encephalopathy. A normal aEEG pattern (continuous normal voltage (CNV) or discontinuous normal voltage (DNV)) AND Sarnat stage 1–2 constituted moderate encephalopathy.

### H-MRS examination

Proton MR spectroscopy studies were carried out on a 3 Tesla Philips Achieva MRI scanner (Philips Medical Systems, Best, The Netherlands), at the MR Research Center of Semmelweis University, as early as the infant reached clinical stability and was suitable for transport. All MR scans were performed between 6th and 96th postnatal hours (median 25th postnatal hour). The Neonatal Emergency & Transport Services of the Peter Cerny Foundation provided the neonatal transport and the critical care, including hypothermia treatment. For the time of the examination, the infants were removed from the incubator and received continuous morphine sedation. In case of intubated infants, skilled personnel provided manual ventilation with an AMBU bag throughout the MR examination. Continuous monitoring of transcutaneous oxygen saturation and capnography was provided for all neonates during the MR scan, using Medrad Veris MR Monitoring System (Bayer Healthcare LLC, Whippany, NJ).

#### Ethical considerations

Patients enrolled did not undergo procedures or interventions for the purposes of the study. Brain MRI and H-MRS are part of routine diagnostic imaging in our unit as a center practice, and are performed on all neonates with suspected moderate-to-severe HIE. Use of these imaging tools aid in confirming the diagnosis, determining the timing of and nature of the hypoxic-ischemic insult (chronic intrauterine or intrapartum), and ruling out other etiologies. Finally, H-MRS measurements are not used to redirect clinical care of infants with HIE.

#### Acquisition protocols

MR spectra were acquired using the PRESS (Point RESolved Spectroscopy) single voxel localization sequence, at echo-times TE = 35 ms, 144 ms and 288 ms, repetition time TR = 2000, number of acquisitions NSA = 128. Duration of scan was approximatively 30 min. The analyzed VOI was a 1 × 1 × 1 cm voxel in the left thalamus of infants, localized based on gradient echo survey images acquired with TE = 5 ms, TR = 75 ms and 30° flipangle.

#### Registered metabolites

The most frequently determined and analyzed metabolites in the H-MRS spectra are N-acetyl-aspartate (NAA), creatine (Cr), choline (Cho), myo-inositol (mIns) and lactate (Lac).

**Fig. 2:**
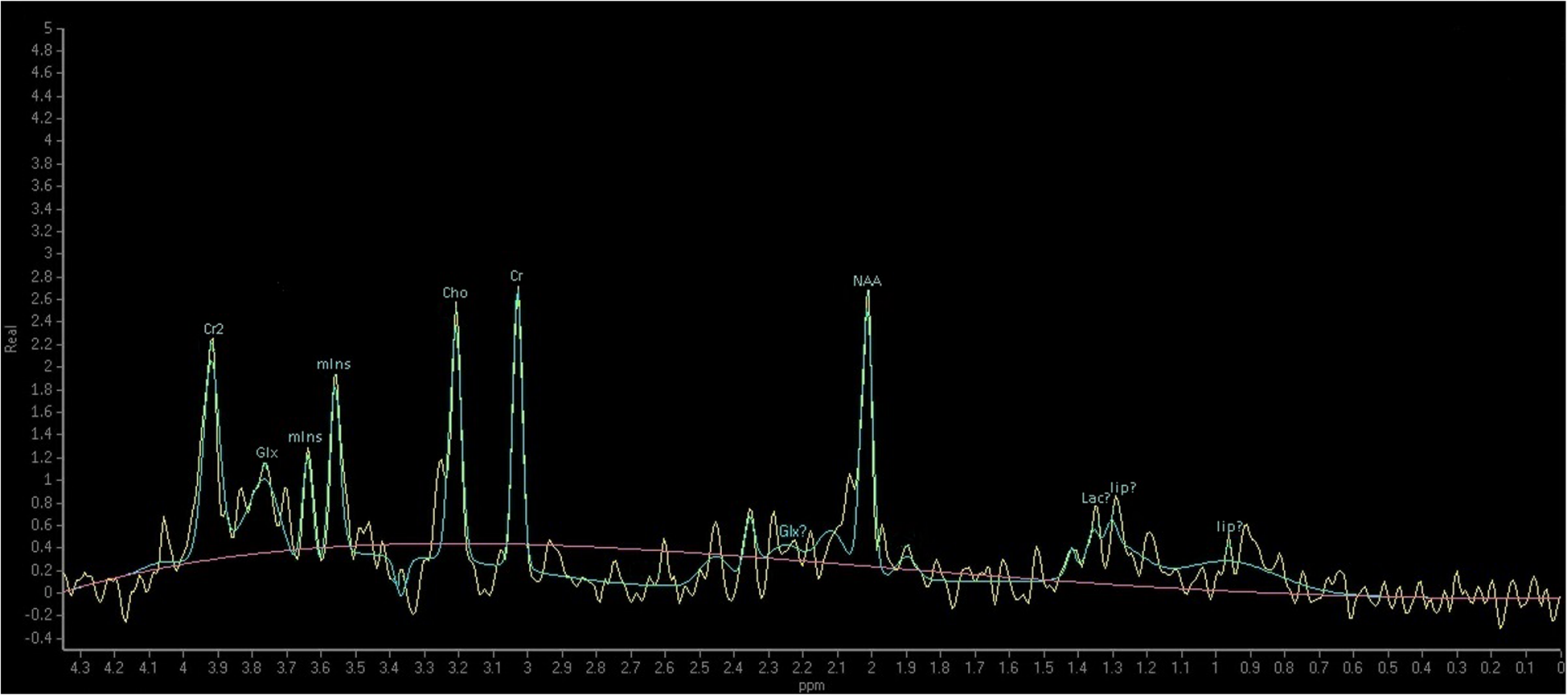
Spectrum acquired by H-MRS at echo time (TE) = 35 ms. The registered metabolites are from left to right: Cr2: secondary creatine peak, Glx: glutamine/glutamate (multiple peaks, here, double peaks), mIns: myo-inositol (double peaks), Cho: choline, Cr: primary creatine peak, NAA: N-acetyl-aspartate, Lac: lactate, lip: lipid (double peaks). In HIE, metabolites considered to have clinical significance are mIns, Cho, Cr, NAA and Lac.? represent low (< 1) signal-to-noise ratio (SNR). NB: at TE = 35 ms, the Lac peak is difficult to differentiate from the overlapping lip peaks

There are different TE optima for the different metabolites, due to their acquisition-dependent signal-to-noise characteristics, e.g. the Lac’s optimum is at TE = 288 ms, while for mI either TE = 35 ms or TE = 144 ms suffices. We recorded peak height, and peak area for all the above-listed metabolites (Fig. 2).

#### MR data analysis

We used the vendor-provided data-processing software on the MR console for analysis without any specific equipment or tool for data-optimization, in order to obtain results applicable to a general clinical setting. In order to reproduce basic, non-research center hospital level circumstances, no data-optimizing equipment or further post-processing methods were used to ameliorate the registered spectra.

Since we did not use absolute quantification protocols due to their high technical requirements, statistical analysis was carried out on all possible ratios of metabolite spectral peak heights and peak areas-under-curve, recorded at same TE. This resulted in the determination of overall 36 metabolite ratios (Table 1).

**Table 1:** List of ratios of peak heights and peak areas of the analyzed metabolites: (NAA: N-acetyl-aspartate, Cho: choline, Cr: creatine, mIns: myo-inositol and Lac: lactate), determined at echo-times TE = 35 ms, 144 ms and 288 ms

TE = 35 ms	TE = 144 ms	TE = 288 ms
• NAA/Cho height and area • NAA/Cr height and area • Cho/Cr height and area • mIns/NAA height and area • mIns/Cho height and area • mIns/Cr height and area	• NAA/Cho height and area • NAA/Cr height and area • Cho/Cr height and area • mIns/NAA height and area • mIns/Cho height and area • mIns/Cr height and area	• NAA/Cho height and area • NAA/Cr height and area • Cho/Cr height and area • Lac/NAA height and area • Lac/Cho height and area • Lac/Cr height and area

To improve the accuracy of our analyses, we excluded metabolite ratios derived from metabolite spectra with signal-to-noise ratio (SNR) below 1, i.e. where noise intensity exceeded signal intensity [23, 24].

### Follow-up

Neurodevelopmental follow-up was measured by Bayley Scales of Infant Development II tool-kit, performed between 18 and 26 months of age by trained personnel, blinded to the H-MRS results. We defined poor outcome as either death (< 28 days of age OR > 28 days associated with HIE) OR moderately/severely delayed development (Mental Developmental Index (MDI) or Psychomotor Developmental Index (PDI) < 70). All other outcomes were considered as good outcome.

### Statistical analysis

Categorical variables are reported as absolute numbers and percentages while continuous variables as mean ± standard deviation or median [25th to 75th interquartile range] depending on the distribution of the parameters. Shapiro-Wilk test was used to assess normality. Categorical variables were compared with the Fisher’s exact test, while continuous variables were compared with the Student t-test or Mann-Whitney U-test for parametric and non-parametric comparisons, respectively.

To select the best metabolite ratios for prognostication, a three-step statistical procedure was implemented. First, we tested the association between the metabolite ratios and outcome. To adjust for multiple testing, we used Bonferroni-correction, that is to say, due to 36 examined metabolite ratios, we considered statistical results significant at *p* < 0.0014 (0.05/36 = 0.0014).

Second, we considered the fact that in the early hours after hypoxic insult, the brain metabolic activity shows extreme variations in time-dependent fashion [25, 26]. Therefore, metabolite ratios measured by H-MRS may also vary significantly depending on timing of data acquisition. Considering that these metabolic changes are still not fully understood and described, we aimed to select metabolite ratios with low or no variability during the first 96 postnatal hours, in order to ensure generalizability of our results for all infants within this period, irrespective of timing of the MR examination. We tested postnatal age-dependence of metabolite ratios using Spearman rank-correlation analysis.

Third, we evaluated the prognostic performance of metabolite ratios using Receiver Operating Characteristics (ROC) curve analysis to establish the potential cut-off-value (corresponding to the highest likelihood ratio of ROC curves), as well as to determine the sensitivity, specificity, positive and negative predictive values of the proposed markers. Moreover, we compared the metabolite ratios as diagnostic tests using the area under the ROC curve (AUC) using the method described by Hanley and McNeil [27].

Demographic, clinical and spectral data were analyzed and plotted using the GraphPad Prism software version 6.0 (GraphPad Software Inc., San Diego, California, USA).

## Results

Fifty-one neonates with moderate-to-severe HIE met the inclusion criteria, and were enrolled in our study. Hypothermia treatment was initiated before the 6th postnatal hour for all 51 neonates, with median [IQR] 2 [1.4; 3.1] hours. Forty-five out of the 51 patients had the H–MRS examination performed while receiving whole-body hypothermia. The remaining 6 patients had the examination done before the initiation or after the completion of hypothermia treatment. Nevertheless, all scans were performed within 96 h of age.

Clinical characteristics and MRI findings of these patients are shown in Tables 2 and 3, accordingly**,** categorized by long-term outcome. Of the 51 patients, 16 infants were considered to have poor outcome, including the 9 patients that died in the perinatal period (i.e. first 28 days), and the 7 patients who had moderately/severely delayed development (Mental Developmental Index (MDI) or Psychomotor Developmental Index (PDI) < 70). Of these 7 patients, 4 infants were diagnosed with cerebral palsy (2 associated with mental retardation and one with epilepsy), 2 had mental retardation and one patient suffered from neuronal hearing loss and epilepsy. None of our patients died between 28 days and the follow-up examination. Good and poor outcome groups only differed significantly in their 5′ and 10′ Apgar scores, as well as occurrence of stage 3 HIE seen on MR images.

**Table 2:** Clinical characteristics of newborns enrolled in the study (*n* = 51)

Variable	Good outcome (*n* = 35)	Poor outcome (*n* = 16)	*p* value
Male sex	19 (51%)	12 (75%)	0.1365
Gestational age (weeks)	39 [38; 40]	38 [37; 40]	0.0536
Birth weight (g)	3261 ± 577	3128 ± 537	0.4379
Apgar 1′	2 [1; 4]	1 [0; 2]	0.0091
Apgar 5′	5 [4; 7]	3 [2; 4]	0.0002*
Apgar 10′	6 [5; 8]	4 [2; 4]	0.0005*
Lowest pH < 1 h of age	7.21 [6.98; 7.28]	7.10 [7.00; 7.20]	0.1643
Highest BD < 1 h of age	14.1 ± 5.9	17.1 ± 4.6	0.0860
Onset of hypothermia (h)	1.8 [1.4; 3.1]	2.1 [1.4; 3.3]	0.6309
Severity of encephalopathy (severe)^#^	31 (89%)	15 (94%)	> 0.9999
Clinical or aEEG seizures (< 24 h)	28 (80%)	14 (88%)	0.7012
Abnormal aEEG pattern (BS, LV, FT)	31 (88%)	12 (75%)	0.2396
aEEG normalization (CNV, DNV) < 72 h	22 (63%)	5 (31%)	0.0681
aEEG normalization time (h)	30 [12; 47]	60 [42; 68]	0.1381
Age at MR scan (h)	25 [14; 49]	30 [16; 54]	0.6625
Abnormalities on MR Imaging (T1/T2 weighted imaging or DWI)	13 (37%)	11 (69%)	0.0681
Death	0	9 (56%)	NA^†^

Data shown as median [IQR], mean ± SD or percentage. Good outcome is defined as both MDI (Mental Developmental Index) and PDI (Psychomotor Developmental Index) ≥ 70 achieved on Bayley II test, poor outcome is defined as either MDI or PDI < 70 OR death (< 28 days of age OR > 28 days of age associated with HIE)

*BD* base deficit, *aEEG* amplitude-integrated electroencephalography, *CNV* continuous normal voltage, *DNV* discontinuous normal voltage, *BS* burst suppression, *LV* low voltage, *FT* flat trace, *DWI* diffusion weighted imaging

* represents significant results surviving Bonferroni-correction (*p* < 0.0014)

# moderate encephalopathy: 6 h normal aEEG pattern (CNV, DNV) AND Sarnat stage 1–2, severe encephalopathy: 6 h abnormal aEEG pattern (BS, LV, FT) OR Sarnat stage 3 [21]

NA^†^ (not applicable) represents statistical significance not applicable as death was included in the definition of the poor outcome group

**Table 3:** Location and severity of observed MR Imaging abnormalities in newborns with good versus poor outcome

	MRI abnormality and good outcome (*n* = 13)	MRI abnormality and poor outcome (*n* = 11)	*p* value
Location of injury			
*Basal ganglia and thalami*	6 (46%)	8 (72%)	0.2397
*Internal capsule*	5 (38%)	6 (54%)	0.6824
*White matter*	5 (38%)	0 (0%)	0.0411
*Cortex*	1 (8%)	1 (9%)	> 0.9999
Severity of injury (MRI score)			
*HIE score 1*	3 (23%)	2 (18%)	> 0.9999
*HIE score 2*	6 (46%)	3 (27%)	0.4225
*HIE score 3*	0 (0%)	8 (72%)	0.0002*

Abnormalities are described as signal intensity abnormality on T1/T2 weighted images, or diffusion abnormality. Severity of injury is described as MR imaging score of HIE [35], assigned by our neuroradiologist blinded to the newborns’ clinical condition

* represents significant results surviving Bonferroni-correction (*p* < 0.0014)

Of the 36 metabolite ratios evaluated in the first 96 postnatal hours for prognostication of good or poor neurodevelopmental outcome, 3 metabolite ratios differed significantly between the good and poor outcome groups, rendering them candidates for further analysis (Table 4): mIns/NAA height (TE = 35 ms), NAA/Cr height (TE = 144 ms) and mIns/Cr height (TE = 35 ms).

**Table 4:** Metabolite ratios differing significantly between the outcome groups (*p* < 0.0014)

Assessed metabolite ratio	value of metabolite ratio (median [IQR])		*p* value
	good outcome (*n* = 35)	poor outcome (*n* = 16)	
mIns/NAA height (TE = 35 ms)	0.534 [0.440; 0.601]	0.780 [0.694; 0.894]	< 0.0001
NAA/Cr height (TE = 144 ms)	0.990 [0.897; 1.096]	0.808 [0.690; 0.863]	< 0.0001
mIns/Cr height (TE = 35 ms)	0.471 [0.387; 0.530]	0.640 [0.528; 0.724]	0.0005

For all other metabolite ratios, see Additional file 1

**Fig. 3:**
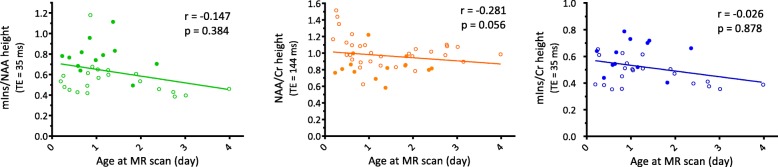
Age-correlation diagrams of the 3 metabolite ratios showing strong association with outcome. Measurements from good outcome group are marked by empty bullet (○), measurements from poor outcome group are marked by circle bullet (●)

Next, we tested the age-dependence of these 3 metabolite ratios during the first 96 postnatal hours among all 51 patients, as it has been described that the brain metabolic activity shows extreme time dependent variations in the early hours after hypoxic insult. We aimed to search for a uniformly detectable metabolite ratio that would be suitable for prognostication any time during the first 4 postnatal days. Assessment of age-dependence did not show significant correlation between either of the 3 metabolite ratios and age at the H-MRS examination. All 3 metabolite ratios showed weak correlation with the timing of the examination, hence might be considered relatively stable during the first 96 postnatal hours (Fig. 3).

**Fig. 4:**
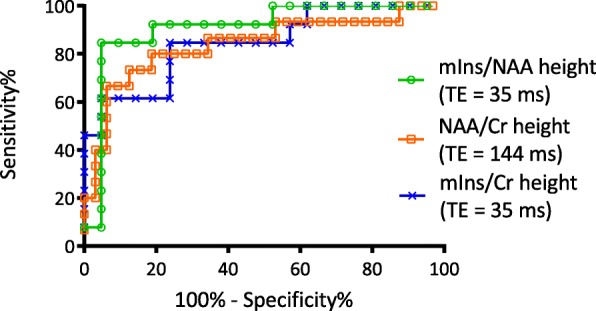
Receiver Operating Characteristics (ROC) curves of metabolite ratios showing weak correlation with age at scan. The area under the ROC curve was 0.9084 for mIns/NAA (TE = 35 ms), 0.8396 for NAA/Cr (TE = 144 ms) and 0.8462 for mIns/Cr (TE = 35 ms) heights (*p* < 0.00001)

Finally, comparing the prognostic performance of these 3 relatively age-independent metabolite ratios, mIns/NAA height at TE = 35 ms had a better discriminative power than NAA/Cr height at TE = 144 ms and mIns/Cr height at TE = 35 ms to identify patients with good versus poor outcome (cut-off-values 0.6798, 0.6274 and 0.7798, respectively, AUC: 0.9084, 0.8396 and 0.8462, respectively, difference between ROC curves *p* < 0.00001). Thus, out of the 36 evaluated metabolite ratios within the first 96 h of age, mIns/NAA height at TE = 35 ms seems to give the best prediction of outcome, with 84.6% sensitivity and 95.2% specificity, irrespective of the timing of the MR examination (Fig. 4 and Table 5).

**Table 5:** Results of Receiver Operating Characteristics (ROC) analysis

Assessed metabolite ratio	Cut-off-value	Area under curve (AUC)	Sensitivity	Specificity	Positive predictive value	Negative predictive value
mIns/NAA height (TE = 35 ms)	0.6798	0.9084	84.62%	95.24%	91.67%	90.91%
NAA/Cr height (TE = 144 ms)	0.7798	0.8396	70.59%	85.29%	75,00%	75,61%
mIns/Cr height (TE = 35 ms)	0.6274	0.8462	61.54%	95.24%	88.89%	80.00%

Difference between ROC curves was significantly different (*p* < 0.00001)

## Discussion

To the best of our knowledge, this preliminary study with a relatively small sample size is the first one that investigated the prognostic accuracy of conventional H-MRS examination performed during the first 4 postnatal days in a group of infants with moderate to severe HIE in the era of hypothermia treatment. We found that myo-inositol/N-acetyl-aspartate height ratio (TE = 35 ms) was the best predictor of neurodevelopmental outcome at 2 years of age. This metabolite ratio proved to have low correlation with age at MR scan during the first 4 postnatal days and showed a specificity of 95.2% and a sensitivity of 84.6% for discriminating between good and poor outcome.

Previously, several studies investigated the prognostic power of H-MRS in asphyxiated neonates using various methods, protocols and equipment [10–19]. Nevertheless, it is problematic to draw an overarching conclusion applicable for the general clinical practice, due to the difference of the methods, findings and conclusions. Indeed, a wide range of H-MRS derived metabolites were suggested as potential biomarkers, e.g. some studies concluded that absolute Lac levels and/or Lac-containing metabolite ratios (Lac/NAA, Lac/Cho, Lac/Cr) were the most accurate in prediction of outcome [11–14, 16–19], while others showed that NAA/Cr, NAA/Cho, absolute NAA and/or Cho levels had promising prognostic powers [10, 11, 15, 17, 18], but only few studies investigated glutamate (Glx) or glutamate-containing metabolite ratios (Glx/Cr) [16], and/or mIns [17].

Interpretation and generalizability of these results are hindered by the fact that there was a marked variability regarding the methods used, some studies applied various data-optimizing software [15, 18] methods for absolute metabolite quantification [14, 15], or special head-coils [14, 17] in order to ameliorate the information acquired from the metabolite spectra. These methods may indeed improve data quality; however, they are not generally applicable in standard clinical settings [20].

Therefore, our intent was to prove the clinical utility of conventional H-MRS sequence with vendor-provided analysis tools in the diagnostic workup of neonatal asphyxial encephalopathy.

Despite their limitations, existing evidence largely supports the use of peak areas as prognostic markers in patients with neonatal encephalopathy. A meta-analysis concluded that deep gray matter Lac/NAA peak area ratio is the most accurate predictor of adverse outcome [28]. Based on Bottomley’s comprehensive review of MR spectroscopy [29] however, without post-processing techniques, the use of peak height and peak area has certain challenges. While peak height provides an acceptable measure for non-overlapping peaks, it is affected by patient motion and inhomogeneous widening of spectrum widths. Peak areas are relatively immune to motion artefacts and spectrum widening. However, since most of the integrated area of a peak resides near its base, noise and overlapping of other peaks can significantly affect the measurements. Taking these factors into consideration, we assessed the prognostic value of both peak heights and peak areas. Based on our findings, it seems that without the use of post-processing, peak height may have an appropriate predictive value and might be useful in the common clinical setting without the use of specialized imaging and post-processing techniques.

We set out to find markers that have similar or possibly even higher value for prognostication than markers published earlier.

To this end, we targeted our investigation on H-MRS scans performed the earliest possible, within 96 postnatal hours, presuming that the earlier the accurate prognostic information, the higher its clinical importance. The majority of the above-listed studies investigated H-MRS scans that were performed significantly later and in a wider range of infant age (3 to 45 days of age) [10, 11, 15–19], with only three papers focusing on early infant ages similar to our study [12–14], all three analyzing considerably small patient cohorts. One of them investigated infants during their first day of life (31 neonates of 4–18 postnatal hours); however, considering the unstable clinical status of many severely asphyxiated infants, this may be unfeasible in the clinical practice [12]. The second paper (11 neonates of 12–48 postnatal hours) concluded that only combined H-MRS and diffusion-weighted imaging is capable of accurate prediction of outcome [13], while the third one (17 neonates of 48–96 postnatal hours) used absolute quantification and a custom-made head-coil to optimize data acquisition [14]. Nevertheless, none of these early-acquisition studies examined neonates while undergoing therapeutic hypothermia.

In addition, although a recent study examined 88 infants with perinatal asphyxia who underwent therapeutic hypothermia, MR scans and H-MRS were acquired only within the first 7 postnatal days [30].

In the era of therapeutic hypothermia, the effect of cooling on brain metabolites is an important issue. Existing evidence suggests that hypothermia increases the clearance of lactate upon cerebral reperfusion [31] and increases overall lactate and myo-inositol levels in the cortex, while increasing the level of taurine and decreasing the level of choline in the thalamus [32]. Even though further studies are needed to outline the hypothermia-induced changes in metabolites detected by H-MRS, these findings suggest that thalamic myo-inositol/N-acetyl-aspartate values are not affected by cooling.

As an essential step in our analysis, we searched for metabolite ratios independent from postnatal age at the MR examination. It is well-known that in the early hours after hypoxic insult, the brain has an extremely dynamic metabolic profile [25, 26], so theoretically, metabolite ratios measured by H-MRS may vary significantly depending on the timing of the MR examination. In addition, the timing of the MR scan is influenced by the clinical stability of newborns. Based on these considerations, the acquisition of a single cut-off value for the proposed biomarker suitable for differentiation between outcomes may be extremely complex, given that the time-dependent metabolite changes are still not fully understood and described. Subsequently, the prognostic markers that vary depending on patient age may show false negative or false positive results, if performed too early or too late in the examined time period, so would require a dynamic range of cut-off-values (cut-off-curve) which calls for considerably larger population and/or repeated measures. Conclusively, until the precise kinetics of brain metabolites are described, the cut-off-value of the proposed prognostic marker should ideally not change with time but should only be determined by severity of encephalopathy and potential outcome. None of the existing studies contemplated the possible postnatal age dependence of the observed metabolites or metabolite ratios. Therefore, we aimed to investigate the stability of metabolite ratios, and found that none of the 3 metabolite ratios associated with outcome showed correlation with timing of the examination in the investigated time window, hence could be potentially independent of postnatal age. We consider the contemplation of the time-dependence of brain metabolites as one of the strengths of our analysis, even though further dependence analyses in repeated measures and larger population are needed to confirm our findings.

Existing evidence suggests that the role of both myo-inositol and N-acetyl-aspartate is complex. Myo-inositol is a pentose sugar, precursor for inositol-derived lipid synthesis and part of the intracellular second-messenger system [33]. To date, studies suggest that myo-inositol could be the breakdown product of abnormal cerebral inositol-polyphosphate metabolism and the cell membrane component myelin [34], implementing that increased myo-inositol levels signal cell death.

N-acetyl-aspartate is the second most abundant amino acid in the brain, functioning as an osmolite with multiple functions, e.g. molecular water pump for neurons to help osmotic regulation, as well as source, storage and transport of acetyl-group, aspartate and amino-nitrogen, for protein and lipid synthesis [33]. Studies suggest that NAA levels decrease after neuronal injury or dysfunction, even in the absence of cell death [34].

Conclusively, neuronal injury induced by hypoxia-ischemia is considered to raise myo-inositol levels and decrease N-acetyl-aspartate levels, thus increasing myo-inositol/N-acetyl-aspartate ratio and providing scientific background for our findings.

It is surprising that none of the lactate-containing metabolite ratios met the strict significance requirements of Bonferroni-correction. One of the reasons for this finding might be the low quality of lactate spectral data. In our measurements, signal-to-noise ratio of lactate peaks were extremely low, with a median [IQR] signal-to-noise ratio of 1.0 [0.7; 1.6] without selection, and 1.6 [1.1; 2.5] after selection based on SNR = 1 criterion. However, low spectral data quality only affected peaks of lactate, since all other metabolites showed significantly more favorable signal-noise characteristics, with a median [IQR] signal-to-noise ratio of 10.8 [8.0; 12.8] for N-acetyl-aspartate, 11.9 [8.8; 14.5] for choline, 11.4 [7.3; 13.9] for creatine and 5.7 [4.8; 7.5] for myo-inositol, reflecting significantly better data quality. Based on these findings, spectral peak of lactate cannot be accurately assessed and interpreted in the general clinical setting and in the absence of post-processing techniques, despite its widespread use in previous studies.

In our study, the volume of interest was a 1 × 1 × 1 cm voxel in the left thalamus. In this cohort, only one patient presented with watershed injury in the left parieto-occipital region, and one patient with widespread cortical lesion. Due to the low prevalence of watershed lesions, we were unable to assess the prognostic value of H-MRS in this type of neuronal injury.

Our study also has a number of limitations. Even though we outlined our methodology to eliminate all possible errors, there are certain points that still might have given way to inaccuracy in our conclusions. First, our study is retrospective in nature, therefore we could not control for factors possibly affecting the findings such as the imaging process and the clinical parameters. This may be considered a limitation compared to a prospective clinical study, where imaging and clinical parameters would have been fine-tuned for the purpose of the study. On the other hand, this could be viewed as a strength from a clinical standpoint, since we had to rely on data that could have been obtained in any MR facility imaging asphyxiated neonates. Therefore, our findings might have more relevance in the general clinical practice. The small sample size of our population is another limitation decreasing the accuracy and reliability of the statistical analysis. The difference between the sizes of the outcome groups (35 good versus 16 poor) might also be considered as a limitation, as our analysis might have been underpowered. Moreover, some may criticize our approach, and may state that all neonates should be examined at the exact same age, which would enable prognostic results to be as accurate as possible. However, considering that infants cannot be assessed before reaching certain clinical stability, this would not be a realistic expectation in the clinical practice.

Obviously, our results must be verified in prospective trials on larger populations and on different MR scanners to corroborate the prognostic power of the proposed H-MRS metabolite ratios.

## Conclusions

In summary, we propose that H-MRS performed before 96 h of age is a potentially promising tool for early prediction of outcome in asphyxiated neonates. The use of H-MRS may add valuable information for the clinicians to assess the severity of the hypoxic insult and potentially utilize additional neuroprotective therapies. Furthermore, our results suggest that even conventional H-MRS might have a high enough prognostic accuracy to be used in routine clinical practice.

## Additional file

Additional file 1: Results of Mann-Whitney test for all metabolite ratios. Data are shown as median [IQR], results were considered significant at *p* < 0.0014 (after Bonferroni correction). (XLSX 10 kb)

## Abbreviations

aEEG: amplitude-integrated electroencephalography
AUC: Area-under-curve
BD: Base deficit
BS: Burst suppression
Cho: Choline
CNV: Continuous normal voltage
Cr: Creatine
DNV: Discontinuous normal voltage
DWI: Diffusion weighted imaging
FT: Flat trace
Glx: Glutamate
HIE: Hypoxic-ischemia encephalopathy
H-MRS: Proton magnetic resonance spectroscopy
Lac: Lactate
lip: Lipid
LV: Low voltage
MDI: Mental Developmental Index
mIns: myo-inositol
MR: Magnetic resonance
MRI: Magnetic resonance imaging
NAA: N-acetyl-aspartate
PDI: Psychomotor Developmental Index
ROC: Receiver Operating Characteristics
SNR: Signal-to-noise intensity
TE: echo-time
VOI: Volume of interest

## References

[1] Kurinczuk JJ, White-Koning M, Badawi N (2010). Epidemiology of neonatal encephalopathy and hypoxic-ischaemic encephalopathy. Early Hum Dev.

[2] Mwaniki MK, Atieno M, Lawn JE, Newton CRJC (2012). Long-term neurodevelopmental outcomes after intrauterine and neonatal insults: a systematic review. Lancet.

[3] Edwards AD, Brocklehurst P, Gunn AJ, Halliday H, Juszczak E, Levene M (2010). Neurological outcomes at 18 months of age after moderate hypothermia for perinatal hypoxic ischaemic encephalopathy: synthesis and meta-analysis of trial data. BMJ.

[4] Rogers EE, Bonifacio SL, Glass HC, Juul SE, Chang T, Mayock DE (2014). Erythropoietin and hypothermia for hypoxic-ischemic encephalopathy. Pediatr Neurol.

[5] Aly H, Elmahdy H, El-Dib M, Rowisha M, Awny M, El-Gohary T (2015). Melatonin use for neuroprotection in perinatal asphyxia: a randomized controlled pilot study. J Perinatol.

[6] Sabir H, Scull-Brown E, Liu X, Thoresen M (2012). Immediate hypothermia is not neuroprotective after severe hypoxia-ischemia and is deleterious when delayed by 12 hours in neonatal rats. Stroke.

[7] The American College of Obstetricians and Gynecologists (2014). Neonatal encephalopathy and neurologic outcome, second edition. Obstet Gynecol.

[8] Douglas-Escobar M, Weiss MD (2015). Hypoxic-ischemic encephalopathy: a review for the clinician. JAMA Pediatr.

[9] Barker PB, Lin DDM (2006). In vivo proton MR spectroscopy of the human brain. Prog Nucl Magn Reson Spectrosc.

[10] Peden CJ, Rutherford MA, Sargentoni J, Cox IJ, Bryant DJ, Dubowitz LM (1993). Proton spectroscopy of the neonatal brain following hypoxic-ischaemic injury. Dev Med Child Neurol.

[11] Groenendaal F, Veenhoven RH, van der Grond J, Jansen GH, Witkamp TD, de Vries LS (1994). Cerebral lactate and N-acetyl-aspartate/choline ratios in asphyxiated full-term neonates demonstrated in vivo using proton magnetic resonance spectroscopy. Pediatr Res.

[12] Hanrahan JD, Cox IJ, Azzopardi D, Cowan FM, Sargentoni J, Bell JD (1999). Relation between proton magnetic resonance spectroscopy within 18 hours of birth asphyxia and neurodevelopment at 1 year of age. Dev Med Child Neurol.

[13] L’Abee C, de Vries LS, van der Grond J, Groenendaal F (2005). Early diffusion-weighted MRI and 1H-magnetic resonance spectroscopy in asphyxiated full-term neonates. Biol Neonate.

[14] Cheong JLY, Cady EB, Penrice J, Wyatt JS, Cox IJ, Robertson NJ (2006). Proton MR spectroscopy in neonates with perinatal cerebral hypoxic-ischemic injury: metabolite peak-area ratios, relaxation times, and absolute concentrations. Am J Neuroradiol.

[15] Boichot C, Walker PM, Durand C, Grimaldi M, Chapuis S, Gouyon JB (2006). Term neonate prognoses after perinatal asphyxia: contributions of MR imaging, MR spectroscopy, relaxation times, and apparent diffusion coefficients. Radiology.

[16] Zhu W, Zhong W, Qi J, Yin P, Wang C, Chang L (2008). Proton magnetic resonance spectroscopy in neonates with hypoxic-ischemic injury and its prognostic value. Transl Res.

[17] Ancora G, Soffritti S, Lodi R, Tonon C, Grandi S, Locatelli C (2010). A combined a-EEG and MR spectroscopy study in term newborns with hypoxic-ischemic encephalopathy. Brain and Development.

[18] van Doormaal PJ, Meiners LC, ter Horst HJ, van der Veere CN, Sijens PE (2012). The prognostic value of multivoxel magnetic resonance spectroscopy determined metabolite levels in white and grey matter brain tissue for adverse outcome in term newborns following perinatal asphyxia. Eur Radiol.

[19] Alderliesten T, de Vries LS, Staats L, van Haastert IC, Weeke L, Benders MJNL, et al. MRI and spectroscopy in (near) term neonates with perinatal asphyxia and therapeutic hypothermia. Arch Dis Child - Fetal Neonatal Ed 2016;fetalneonatal-2016-310514. 10.1136/archdischild-2016-310514 27553589

[20] Wilkinson D (2010). MRI and withdrawal of life support from newborn infants with hypoxic-ischemic encephalopathy. Pediatrics.

[21] Azzopardi DV, Strohm B, Edwards a D, Dyet L, Halliday HL, Juszczak E (2009). Moderate hypothermia to treat perinatal asphyxial encephalopathy. N Engl J Med.

[22] Shalak LF, Laptook AR, Velaphi SC, Perlman JM (2003). Amplitude-integrated electroencephalography coupled with an early neurologic examination enhances prediction of term infants at risk for persistent encephalopathy. Pediatrics.

[23] Blüml S (2013). Magnetic resonance spectroscopy: basics. MR spectroscopy of pediatric brain disorders.

[24] Holmes D. Basic practical NMR concepts: a guide for the modern. Laboratory. 2004:1–42.

[25] Vannucci RC, Towfighi J, Vannucci SJ (2004). Secondary energy failure after cerebral hypoxia-ischemia in the immature rat. J Cereb Blood Flow Metab.

[26] Hassell KJ, Ezzati M, Alonso-Alconada D, Hausenloy DJ, Robertson NJ (2015). New horizons for newborn brain protection: enhancing endogenous neuroprotection. Arch Dis Child Fetal Neonatal Ed.

[27] Hanley JA, McNeil BJ (1983). A method of comparing the areas under receiver operating characteristic curves derived from the same cases. Radiology.

[28] Thayyil S, Chandrasekaran M, Taylor A, Bainbridge A (2010). Pediatrics.

[29] Bottomley P (1991). The trouble with spectroscopy papers. Radiology.

[30] Alderliesten T, De Vries LS, Staats L, Van Haastert IC (2017). Arch Dis Child Fetal Neonatal Ed.

[31] Lei H, Peeling J (1998). Effect of temperature on the kinetics of lactate production and clearance in a rat model of forebrain ischemia. Biochem Cell Biol.

[32] Chan KWY, Chow AM, Chan KC, Yang J, Wu EX (2010). Magnetic resonance spectroscopy of the brain under mild hypothermia indicates changes in neuroprotection-related metabolites. Neurosci Lett.

[33] Brighina E, Bresolin N, Pardi G, Rango M (2009). Human fetal brain chemistry as detected by proton magnetic resonance spectroscopy. Pediatr Neurol.

[34] Macrì MA, D’Alessandro N, Di Giulio C, Di Iorio P, Di Luzio S, Giuliani P (2006). Regional changes in the metabolite profile after long-term hypoxia-ischemia in brains of young and aged rats: a quantitative proton MRS study. Neurobiol Aging.

[35] Barkovich AJ, Hajnal BL, Vigneron D (1998). Prediction of neuromotor outcome in perinatal asphyxia: evaluation of MR scoring systems. Am J Neuroradiol.

